# Historical and Archaeogenomic Identification of High-Status Englishmen at Jamestown, Virginia

**DOI:** 10.15184/aqy.2024.75

**Published:** 2024-08-13

**Authors:** Douglas W. Owsley, Karin S. Bruwelheide, Éadaoin Harney, Swapan Mallick, Nadin Rohland, Iñigo Olalde, Kathryn G. Barca, Andrew J. Ramsey, Deborah A. Hull-Walski, William M. Kelso, Jamie E. May, Nasreen Broomandkhoshbacht, Matthew Ferry, Ann Marie Lawson, Megan Michel, Jonas Oppenheimer, Kristin Stewardson, Fatma Zalzala, Vicki E. Simon, David M. Givens, Michael D. Lavin, David E. Reich

**Affiliations:** 1 Department of Anthropology, National Museum of Natural History, Smithsonian Institution, Washington, D.C., USA; 2 Department of Organismic and Evolutionary Biology, Harvard University, Cambridge, USA; 3 Department of Genetics, Harvard Medical School, Boston, USA; 4 The Max Planck-Harvard Research Center for the Archaeoscience of the Ancient Mediterranean, Cambridge, USA; 5 Department of Human Evolutionary Biology, Harvard University, Cambridge, USA; 6 Howard Hughes Medical Institute, Harvard Medical School, Boston, USA; 7 Broad Institute of MIT and Harvard, Cambridge, USA; 8 Ikerbasque-Basque Foundation of Science, 48009 Bilboa, Spain; 9 BIOMICs Research Group, Department of Zoology and Animal Cell Biology, University of the Basque Country UPV/EHU, 01006 Vitoria-Gasteiz, Spain; 10 Jamestown Rediscovery (Preservation Virginia), Williamsburg, USA

## Abstract

Ancient DNA (aDNA) data are reported for two human skeletons buried within the chancel of the 1608–1616 church at the North American colonial settlement of Jamestown, Virginia. The men are suspected kinsmen of the colony’s first Governor, Thomas West, 3^rd^ Baron De La Warr based on archaeological, osteological, and documentary evidence. Genomic analyses of these men, Sir Ferdinando Wenman and Captain William West, identify a shared mitochondrial haplogroup, H10e, inferring maternal relatedness. In this unusual case, aDNA prompted further historical research that led to the discovery of illegitimacy, an aspect of identity omitted, likely intentionally, from genealogical records.

## Introduction

**Figure 1.**
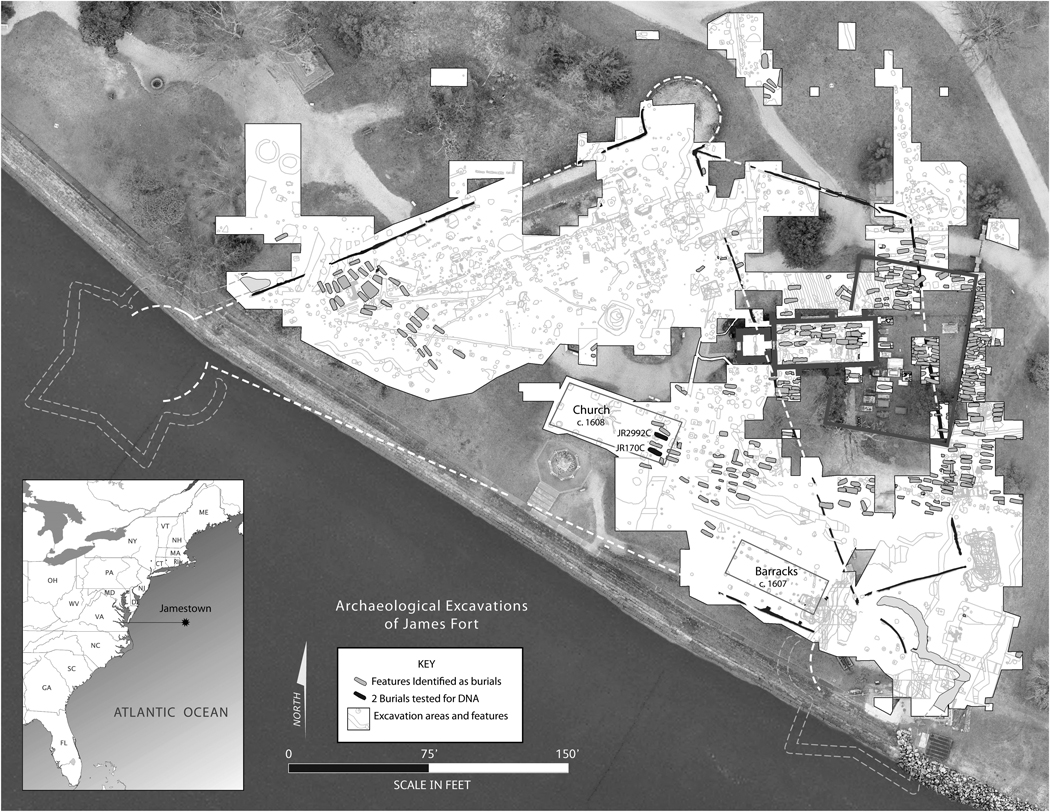
Map of Jamestown Island (North America) with the triangular palisade of James Fort; the 1608 church containing burials in the chancel (JR170C and JR299C highlighted).

In 1994, Jamestown Rediscovery archaeologists uncovered buried evidence of the 1607 James Fort (Figure 1). At this early North American English settlement, known as Jamestown or James City, are the material remains of the beliefs and rituals maintained by the immigrants that arrived in the colony, including evidence of their early churches. Excavations from 2010 through 2019 uncovered remnants of the 1608 church, the settlement’s second, and the overlapping foundations of Jamestown’s subsequent third, fourth, and fifth churches, built around 1617, 1647, and ca. 1680 (post Bacon’s Rebellion), respectively (Givens et al. 2020; Givens et al. 2016; Kelso 2017). Within the bounds of these structures human remains have been encountered and recent research has greatly expanded what is known about the lives of these individuals.

The years-long, integrative approach applied to the study of the human remains recovered from Jamestown merges various data sources culminating in bio-histories that sometimes align with recorded names of the deceased. As an example, the skeleton of an adult male buried outside of James Fort in a coffin with a captain’s leading staff is believed to be that of Bartholomew Gosnold, champion of the Virginia colony venture, whose death and burial following a brief illness were documented in August 1607 (Kelso 2006). In other cases, the context of the bones and burials—as well as associated artifacts and signs of trauma—parallel historic references to Jamestown events and their unnamed victims, including a female whose skeletal markers of survival cannibalism align with purported violent events that occurred at the settlement during the starving time winter of 1609–1610 (Horn et al. 2013; Kelso 2017).

In 2013, four aligned graves were excavated from the chancel area of the 1608 church, centrally located within James Fort. Burial within the chancel, the eastern part of an Anglican church, signifies a particularly high degree of status (Seeman 2010). Historical information on ranking colonists whose deaths coincide with the brief use of the church (ca. 1608–1616), matched with osteological and archaeological data, identified these men as Reverend Robert Hunt (1569–1608), Captain Gabriel Archer (1575–1609), Sir Ferdinando Wenman (also spelled Weyman or Wainmen) (1576–1610), and Captain William West (about 1586–1610) (McCartney 2007; Remington 2014; Givens et al. 2016; Kelso 2017).

This study reports on the ancient DNA (aDNA) analysis of two of these men: Sir Ferdinando Wenman and Captain William West. Both are suspected kinsmen of the colony’s first Governor, Thomas West, 3^rd^ Baron De La Warr. Initially, the study sought to retrieve Y chromosome DNA of the West lineage through Captain William West, who bore the family surname. The intent of this approach was to secure reference data for comparison to future found remains potentially matching with the identity of Thomas West, Governor De La Warr, who died at sea in 1618 during his return voyage to the colony. His burial location is unknown; however, the body may have been brought to Jamestown and buried (Kelso 2017). But, before Captain West’s paternal DNA could be used in this regard, his parentage needed to be confirmed, as it was not clearly defined in the documentary record. Therefore, the study also had the objective of exploring the familial relationship between William West and Wenman, whose well-documented genealogy showed him to be a cousin of Thomas West, 3^rd^ Baron De La Warr.

Due to poor bone preservation, the possibility that this study would recover DNA and yield results was uncertain. However, analysis of this burial pair demonstrates that aDNA can clarify interpretations of identity even for poorly preserved remains. In fact, the aDNA proved to be a critical component in identifying illegitimacy for one of the men and explained why so little was recorded for this presumably high-status individual. The significance of the genomic data would not have been realized, however, without a multidisciplinary approach to the identification. This study demonstrates the use of aDNA in such investigations of archaeological remains, and underscores the role of kinship in colonial settlement, particularly within the context of late 16^th^-early 17^th^ century cultural norms.

### Individuals JR2992C and JR170C

**Figure 2.**
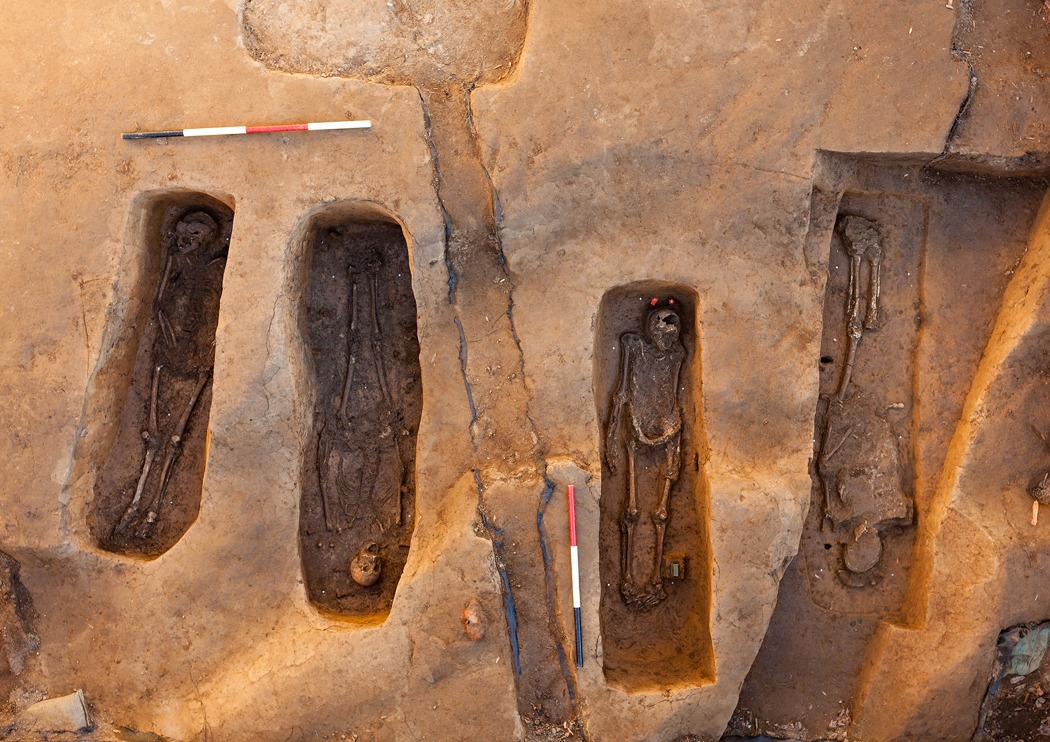
Four aligned graves in the chancel area of the Jamestown 1608 church (ca. 1608–1616). Evidence including shared orientation and coffin style identify the second (JR2992C) and fourth (JR170C) men from the left as possible members of the prominent West family.

Of the four individuals buried in the chancel area of Jamestown’s 1608 church (Figure 2), two (JR2992C and JR170C) had matching coffin styles, a rarely used anthropomorphic form. Similarities in shape and the high quality of the nails used for their construction suggested that the same skilled carpenter built both coffins within a relatively brief period. This evidence, and the estimated ages at death for the two men, (OSM 1), match what is known about Sir Ferdinando Wenman and Captain William West (Jamestown Rediscovery 2016; Kelso 2017).

Wenman (JR2992C), the first English knight to die in America, was a first cousin of Thomas West, 3^rd^ Baron De La Warr. Wenman was related both by blood and marriage to highly prominent families in England during the Elizabethan period (Remington 2014). In contrast, the lineage for Captain William West (JR170C) is obscure. He is referenced in one account as Thomas West’s nephew, in a second as a “kinsman” (Kelso 2017:175), and, based on a genealogical review, seemed more likely to be his younger uncle (Remington 2014). Wenman came to Virginia in June 1610 as a thirty-four-year-old experienced military officer and investor in the Virginia Company. He arrived with Thomas West and Captain William West, who was a man estimated to be in his early twenties. By July or August of 1610, Wenman was dead. Captain West died in a conflict with Native Americans around this same time. Their formal interments in the church chancel were likely personally directed by Thomas West, Baron De La Warr.

Although the life of Sir Ferdinando Wenman is relatively well documented, almost all that is known of Captain West comes from George Percy’s “*A Trewe Relation,”* a first-hand account of the events that took place at Jamestown between the 1609 Bermuda shipwreck of the *Sea Venture* and his departure from Virginia in 1612 (Tyler 1921). Percy first mentions William West on 9 August 1610 when William was to signal a raid against local Indigenous groups by firing his pistol into the air. Baron De La Warr later (no date specified) traveled up-river to the falls near modern-day Richmond, Virginia to construct a fort. Thomas West survived this journey, but among those Percy recorded as dead was “his Kinsman Capte: William Weste” (Tyler 1921).

The skeleton identified as JR170C represents a male in his early twenties. Of note was the skeleton’s unusually high lead level, measured in parts per million by inductively coupled plasma mass spectrometry (ICP-MS) (Little et al. 2014). This young man’s lead value was the second highest of the four men buried in the chancel; Wenman had the highest. Lead exposure has been used as a potential marker of economic and social status, as individuals who consumed foods served on costly pewter and lead-glazed wares accumulated higher amounts of the heavy metal in their bones (Aufderheide et al. 1981, 1985; Aufderheide 1989). Also found with JR170C were remnants of an elaborate silver-fringed and spangled silk military sash, an article of clothing signifying a rank of Captain (Givens et al. 2016; Kelso 2017).

William West’s specific relationship to Thomas West, 3rd Baron De la Warr, and to Ferdinando Wenman was unclear. William could not have been Thomas’ nephew as none of his brothers were old enough to have had a child of William’s age. He was not a brother and did not appear to be a cousin as Thomas’ father, Thomas the 2^nd^ Barron De La Warr, only had three sisters listed in the heraldic visitation from the period, and none were identified as mother to William (Rylands 1913; Tyler 1924). His identification as a kinsman to Thomas West denotes a relationship that was known at the time but has since been obscured by history.

### aDNA Analysis

Genomic data were generated from the petrous portion of a temporal bone from JR2992C and a mandibular molar from JR170C (OSM 2, Table S2). Both skeletons exhibited relatively poor DNA preservation, with a total of 76,449 of the ~1.2 million targeted autosomal single nucleotide polymorphism (SNP) positions sequenced for individual JR2992C, and only 12,657 targeted SNPs sequenced for individual JR170C (Table 1). Despite poor DNA preservation, coverage was sufficient to assign both individuals to mitochondrial haplogroup H10e (Weissenstiner et al. 2016). The H mitochondrial lineage is commonly observed throughout much of West Eurasia, occurring at the highest frequencies in present-day Western Europe, where it accounts for over 40% of all mitochondrial DNA lineages (Torroni et al. 2006; Brotherton et al. 2013). Within them, the relatively rare H10e haplogroup has been observed throughout Europe in multiple ancient individuals, including in two medieval individuals from Finland (Carvalho et al. 2016; Översti et al. 2019).

Crude Y chromosome haplogroup assignments were obtained, with JR2992C assigned to haplogroup I1 and JR170C assigned to haplogroup F. Although haplogroup I falls within the more basal haplogroup F, there was sufficient resolution (based on alleles observed at SNPs CTS7593 and PF3660) to confidently exclude the possibility that JR170C would also be assigned to this more specific haplogroup if more information were available. These results indicate that the two individuals do not share a recent paternal line ancestor (e.g., a father, paternal line grandfather, or great-grandfather).

Despite low coverage across the nuclear genome for these two individuals, it was sufficient to also conclude that they are unlikely to be 1^st^-degree relatives (Olalde et al, 2019). The inferred relatedness coefficient of 0.12 is most consistent with a 3^rd^-degree relationship (such as that shared by first cousins). However, the 95% confidence interval associated with this estimate ([−0.049, 0.280]) is very large. Therefore, although not probable due to their shared mtDNA haplogroups, it is possible that the men share no close genetic relationship.

**Figure 3.**
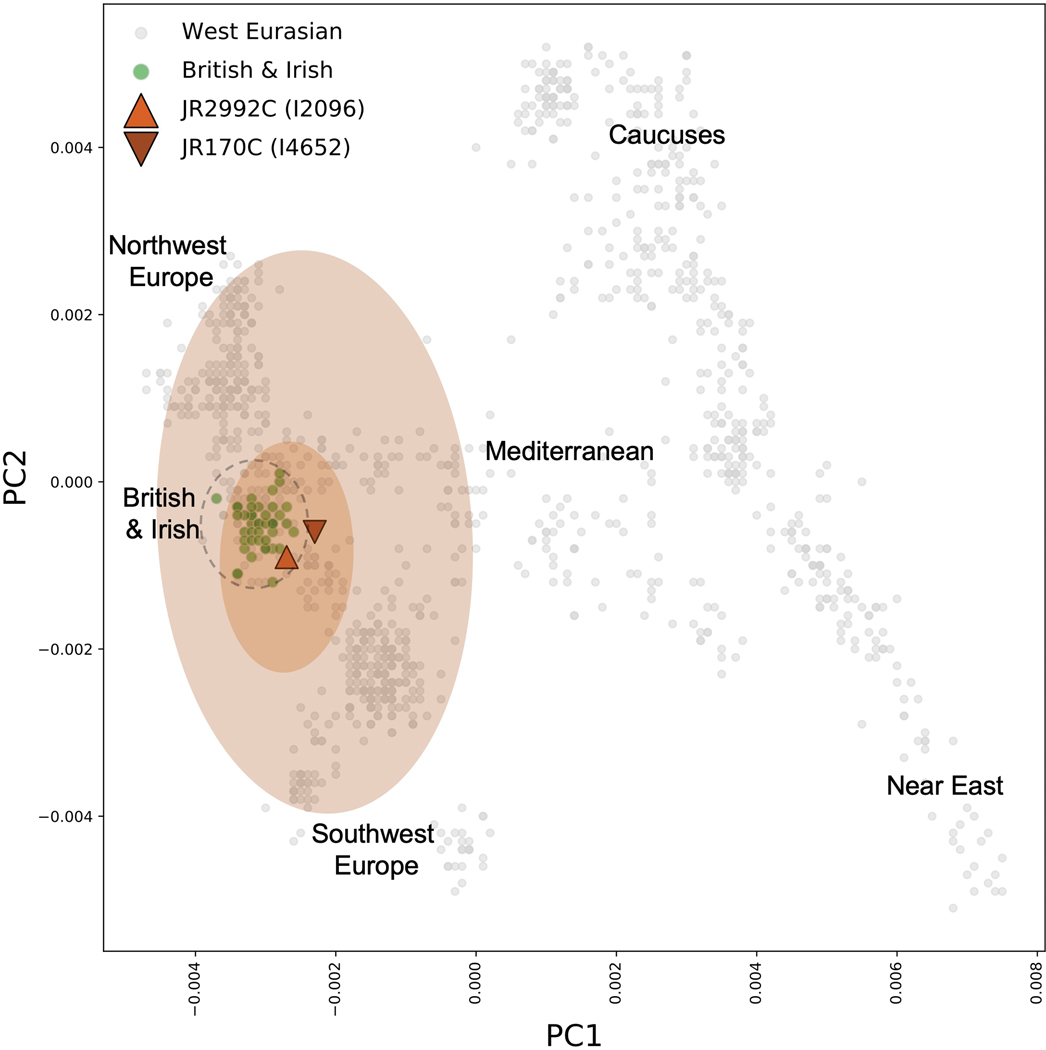
Broadly Western European ancestry detected in JR2992C and JR170C. Principal component analysis of 1,320 present-day individuals from 66 populations from Europe and the Near East from the Human Origins dataset. All present-day individuals are shown in grey. The two Jamestown individuals of European ancestry (JR2992C and JR170C) are projected.

When the genomes of the two individuals are projected onto a Principal Component Analysis (PCA) plot commonly used to localize West Eurasian ancestry, the Jamestown men cluster with those of known British ancestry (Figure 3). However, large 95% confidence intervals are observed around these inferred positions, particularly for individual JR170C. Therefore, based on the small amount of autosomal DNA data that could be obtained from these two men, caution is advised when interpreting the results of any additional analyses performed on these genetic data.

#### Deciphering the familial relationship between JR2992C and JR170C

While DNA preservation of JR2992C and JR170C was too poor to use genome-wide data to conclusively state whether the two individuals share a close genetic relationship (Olalde et al. 2019), the assignment of both individuals to mitochondrial haplogroup H10e shed light on the possible nature of kinship between them. Mitochondrial DNA is inherited along the maternal lineage, meaning that individuals with a shared mitochondrial haplogroup are related through their maternal ancestors at some point in the past. Haplogroup H10e emerged between 2,500–7,000 years ago (Behar et al. 2012), and therefore the timing of the most recent shared maternal ancestor cannot be determined through analysis of the mitochondrial DNA alone. However, these two men were buried in the 1608 church chancel a few feet apart and historical comments refer to them as kinsmen. Haplogroup H10e is comparatively rare in the United Kingdom, having been found in only two of 3,594 individuals in a recent study (Fleskes et al. 2019). Given that one of the individuals carried H10e, the probability that the other would also carry it if they were not close maternal relatives would be 0.056% (i.e., 2/3594). This is such a small probability that it can be concluded with some certainty that they are close maternal relatives.

Based on his surname, it was originally hypothesized that Ferdinando Wenman and Captain West were related through a shared paternal connection that extended back to William West (the elder), 1^st^ Baron De La Warr—a connection that would not have involved a shared maternal lineage. Historical and genealogical research established that William West the elder was born about 1532 and died in 1595 without leaving a known will or any testamentary evidence for a son named William (Remington 2014). William the elder had two wives: Elizabeth Strange whom he married ca. 1554, followed by a second marriage after 1579 to Anne Swift. William and Elizabeth Strange had three daughters, Jane, Mary, and Elizabeth, and one son, his heir, Thomas West, 2^nd^ Baron De La Warr. If the elder William had a surviving son named William, he would have been born to the second wife, Anne. And yet, Anne and the elder William had no recorded children. In addition, Captain William West’s year of birth appears to have been around 1586, based on suspected matriculation into Cambridge University in 1598 at the age of 12 (Remington 2014). If this year of birth is valid, then Anne would have been more than 50 years old at the time of his birth, typically past childbearing age.

Historical research revealed that before leaving for Virginia in 1610, William West the younger left a nuncupative (oral) will that was proved in 1616 in the Prerogative Court of Canterbury which handled the wills of larger English estates at the time. William bequeathed his possessions to Mary Blount (Blunt) wife of Richard Blount and daughter of William West the elder. Whatever the parentage of Captain West, it seems clear that Anne Swift West did not have a close relationship with him after her second husband, William West, 1^st^ Baron De La Warr, died, although Mary Blount may have. The will, and especially the genomic evidence, renewed historical and genealogical research focused on the life histories of William the elder’s daughters and their progeny.

**Figure 4.**
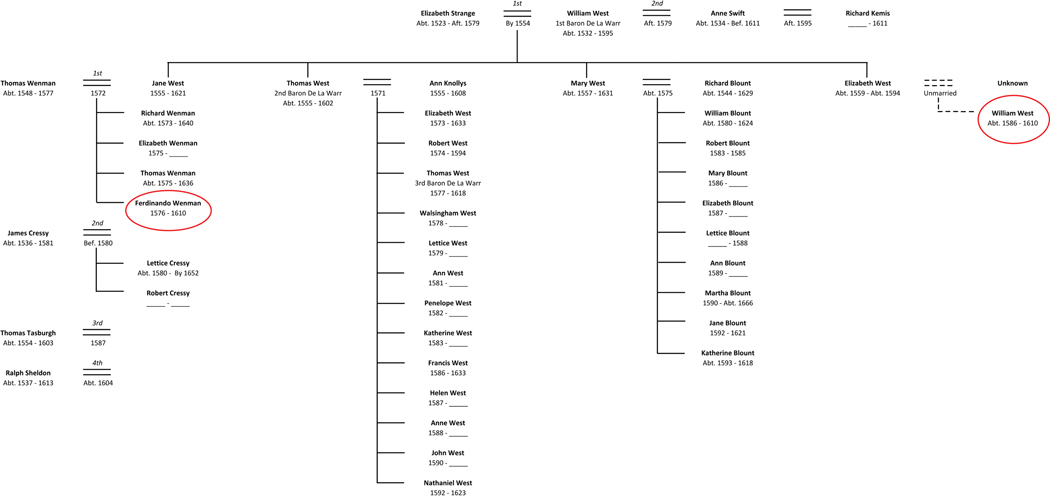
Three generations of the family tree of William West, 1^st^ Baron De La Warr.

Married daughters Jane West Wenman and Mary West Blount had multiple recorded births (Figure 4), and furthermore, Mary had a baby born about the same time that William West the younger seems to have been born. The youngest daughter Elizabeth West had no recorded marriage or children. The known genealogical information failed to identify William’s mother.

Resolution of William’s maternity was eventually found in a court pleading from 1616. *Blount v. Abbot* (1616, on file in the National Archives of the United Kingdom) states that William West the younger had been raised by Mary Blount on behalf of her unmarried, deceased sister, Elizabeth. This explains William’s choice for naming Mary Blount as the beneficiary of his will. When Elizabeth died as a resident in her father’s household, she left her jewels to the young William for his care. After the death of William the elder, his second wife, Anne Swift, took custody of the jewels. After Anne’s death, the jewels moved into the estate of her third husband. After Captain William West’s death, his aunt, caregiver, and will beneficiary Mary Blount attempted to recover the inheritance. Two excerpts from *Blount v. Abbot* clarify these complex relationships.

#### Excerpt 1 – Speaking about Elizabeth West and her relationship to William West:

“And soe being possessed & lying in extremetie of sickness, being the sickness where so she shorlie after dyed and having one onely Cozen or friend (named the said William West) to whom she much desired to have or command the said jewells, chaynes and ornaments, for his better maintenance and preferment in living, he being then very young did by way of bequest or last will verballie Commit the said jewelles, Chanis, and ornaments, to one William West the elder to and for the use, behoofe & maintenance of the said William West the younger.…”

#### Excerpt 2 – The oratrix, Mary West Blount, had cared for William West:

“…William West the younger made his last will and testeament and thereby made your said oratrix executrix & died, after whose decease your said Oratrix lawfullie proved the said will according to his majesties…lawes of his Realme, and during the life of the said William West The younger did maintaine him in …meate, drink, apparel, and other necessearyes by reason the said William West the elder did neglect to performe the trust in him reposed by the said Elizabeth…”

Despite the record stating that William the younger was the cousin of Elizabeth, there is no known family relationship indicating that this was literal. Instead, the statement that William (who was a small child at the time) was her “one onely Cozen or friend” seems to indicate in sanitized, non-incriminating language that William the younger was in fact her child. The coded language and absence of formal documentary recognition of William as her son supports the illegitimate nature of his birth. It also explains why Elizabeth never married, as bearing a child out of wedlock would have likely rendered her unmarriageable, isolated her socially, and caused her considerable shame (Macfarlane 2002). This could also explain why William was given his first name and kept the West surname.

Studies of illegitimacy in preindustrial England document a peak in its occurrence during the late 16^th^ century and early 17^th^ century, a period of converging socio-economic factors and high mortality from recurrent plagues (Cummins, Kelly and Ó Gráda 2016). While studies of illegitimacy show higher rates (often biased based on available records) in communities experiencing poverty and immigration (Scott and Duncun 1997), this study revealed its occurrence within a prominent English family of this era. What can be learned from this?

The study shows that identifying illegitimacy in families of wealth can be difficult as these relationships were often excluded from recorded lineages. The poor had less choice or motivation to hide such births as aid was often sought from the church or court. Harsher treatments and attitudes related to illegitimate birth were rendered to the poor as a result. As Walter King summarized in his review of punishment for bastardy in early 17th-century England, “…it was pauper bastardy and not bastardy per se which was so intolerable” (1978:134). Parents or families (aunts, uncles, or grandparents) financially able to care for the illegitimate child suffered less, although the stigma of birth outside of marriage remained for both family and child. Even the wealthy were not immune to the economic implications of illegitimacy, however, and it seems fitting that Captain West’s story ends in a court case over property linked to his care in childhood.

Another commonality in cases of illegitimacy is of note - departure from one’s natal community. Parents of illegitimate children often moved from the community of birth to hide the child’s status (Low 1989). The adult Captain West likely understood the personal advantages of leaving England and partaking in the Jamestown venture. Since he arrived accompanied by two older cousins, it can be assumed that Captain West’s family also likely encouraged his departure to a place offering greater economic and social opportunities.

It is acknowledged that the extent to which illegitimacy affected Captain William West during his brief life remains mostly unknown. However, his narrative, as revealed through genetics and archival research, documents one prominent 17^th^-century English family’s actions, if not attitudes, towards illegitimacy. The West family had the economic means to care for Elizabeth and her child and chose to do so. Elizabeth remained part of her father’s household until her death. Afterwards, care for William was assumed by his maternal aunt. Although his grandfather neglected to provide for him as his mother wished, West was able to receive an education followed by military service. Military service was a common option for younger sons and distant relations of landed families who were often promised smaller portions of their family’s estates. They sometimes entered the military or made investments into other ventures to better secure their standards of living. For both William West and Ferdinando Wenman, their connections to the West family helped them to obtain positions in the new colony under their cousin, Governor Thomas West. Although their prospects were cut short, both received burials in places of honor within the church at Jamestown. Such an honor would likely not have been extended to Captain William West in England.

## Conclusion

This study is the first to demonstrate that aDNA is a tool in identifying not only ancestry, but historical cases of illegitimacy in high-status 17^th^-century families. Based on the results of the aDNA analysis, extensive genealogical and historical research was conducted to untangle the relationship between Captain William West and Ferdinando Wenman, particularly along their maternal lineage. The record insinuates that William the younger was the illegitimate son of Elizabeth, the unmarried daughter of William West, 1^st^ Baron De La Warr. Jane, Mary, and Elizabeth, as daughters of Elizabeth Strange would have shared the same mitochondrial haplogroup. Ferdinando Wenman and Captain William West, as children of Elizabeth Strange’s daughters would also share that same mitochondrial haplogroup.

aDNA data reported for these two men reveals a previously unrecorded aspect of identity for one, and alters initial perceptions held by researchers regarding his parentage. Despite the study’s failure to secure precise paternal reference data for future comparison with found remains, the DNA findings are appreciated for the clarity they provide to identification, especially within the context of other types of data (i.e., archaeology, skeletal biology, chemical analysis, genealogy, and historical primary source documents). This study reinforces the need for multidisciplinary approaches to better answer questions of not only *who* was involved in colonization, but possibly, *why.*

### Ethical Considerations

The bioarchaeological investigation at Jamestown was conducted in coordination with the Virginia Department of Historic Resources and in accordance with Section 10.1–2305 of the *Code of Virginia* regarding the excavation of human remains from archaeological contexts. The genetic work was conducted in accordance with rigorous guidelines for the ethical analysis of ancient DNA (Alpaslan-Roodenberg et al. 2021). This study was performed in consultation with Preservation Virginia and the Colonial National Historical Park. Bioarchaeological research at Jamestown is supported and endorsed by the Jamestowne Society, which represents genealogical descendants of the colonists to Virginia.

## Supplementary Material

Online Supplementary Materials

## Figures and Tables

**Table 1.** aDNA sample information.

Individual ID	Lab ID	Skeletal element sampled	Coverage on autosomal targets	SNPs hit on autosomal targets	Sex	mthNA haplogroup	mthNA match to consensus rate (%)	Y-chromosome haplogroup*	Damage rate in first nucleotide on sequences overlapping 1240k targets
JR2992C	I2096	petrous	0.069	76,449	M	H10e	97.95 ± 0.98	I1	0.260
JR170C	I4652	second molar	0.011	12,657	M	H10e	99.75 ± 0.13	F	0.112

* Y-Chromosome haplogroup calls made for individuals with less than 100,000 SNPs should be interpreted with extreme caution due to low coverage.

## Data Availability

The aligned sequences are available through the European Nucleotide Archive under accession number **TBD**. Genotype datasets used in the analysis are available at https://reich.hms.harvard.edu/datasets.
