# Supplementary material for: Historical and Archaeogenomic Identification of High-Status Englishmen at Jamestown, Virginia
Source: Antiquity. Author manuscript; Available in PMC 2024 Dec 18. (PMC11654903; doi:10.15184/aqy.2024.75)
Supplement: Online Supplementary Materials [file NIHMS2034812-supplement-Online_Supplementary_Materials.docx]

**Historical and Archaeogenomic Identification of High-Status Englishmen at Jamestown, Virginia**

**Online Supplementary Materials**

These supplemental materials provide detailed data related to the bioarchaeological and genetic analysis of the two skeletons referenced in this paper. OSM1 presents the results of previously conducted bioarchaeological investigations of the two skeletons. These studies followed standard data collection practices (Buikstra and Ubelaker 1994) and included isotopic testing following methods described by France *et al.* (2019) and Ubelaker & Owsley (2003). The heavy metal content in the bones was measured using inductively coupled plasma-mass spectrometry (ICP-MS) techniques as described in Little et al. (2014). Details of the process of genomic assessment are presented in OSM2 with Table S3 providing expanded information for both individuals.

**OSM1: Field and Lab Observations of JR2992C and JR170C**

**JR2992C**

This burial was second from the north end of the Chancel of the 1608 James Fort church (Givens et al. 2016). Although in use by 1608, recent research suggests church construction began in the winter of 1607 (Delano 2022). An adult male was buried in a supine position in an anthropomorphic coffin with the head in the west-end of the grave. This is one of only two excavated Jamestown burials in this style of coffin that has a box-like compartment framing the head, wider at the shoulders and narrower at the feet. The southernmost burial (JR170C) has the same style of coffin. The only associated artifacts are large nails used for the coffin’s construction. The cranium is face-up and slightly inclined to the right. The arms are along the sides of the body. Both forearms have slight pronation and hand bones lie on the sides of the pelvis. The knees and ankles are close together.

The individual is not markedly robust. The crown to heel measurement is 170 cm; breadth estimated at the shoulders is 33.5 cm; at elbows 46 cm; across the knees 19.5 cm; and across the ankles, 15.5 cm.

Bone preservation is poor. The skeleton is represented by a broken cranium, including a nearly complete frontal, complete parietals, a nearly complete occipital, temporals, and partial maxillae. A nearly complete mandible is also present. The postcranial skeleton is partially represented by a right clavicle, fragments of the innominates, mostly complete shafts of the humeri, the proximal end of the right ulna diaphysis, a small fragment of the right radius, femora missing their necks and distal fourths, a left tibia missing its proximal end and distal third, a right tibia shaft that is mostly complete but missing its ends, and the distal third of the right fibula. At least three cervical vertebrae were recovered (C1, C2, C4), along with fragments of thoracic and lumbar vertebrae. Bones of the feet are present, but severely degraded.

The right maxillary dental arcade is complete, and all teeth are present in their sockets
except the central incisor, which is missing. The right lateral incisor has one hypoplastic line. The right maxillary first molar has a periodontal abscess linked to complete carious destruction of the right second molar crown. A small perforation in the floor of right maxillary sinus indicates this abscess had ruptured into the sinus. The left maxillary dentition is represented by four loose teeth and four teeth in their sockets. The left first premolar has an interproximal cavity. Adjoining surfaces of the left second and third molars each have cervical cavities with root involvement. The mandibular dentition is complete except for antemortem loss of the left first premolar; its socket has resorbed. The right mandibular third molar has a small cavity. Moderate calculus deposits are present with slight to moderate alveolar resorption, most notably for the right maxillary molar.

Age

Several features suggest an age of 35 to 39 years. The endocranial and ectocranial sutures are mostly obliterated. Only the coronal suture exhibits slight retention of the suture line on the outer table. Shallow pacchionian depressions are present on the inner table. The largest depression on the left parietal, near the sagittal suture, measures 4.3 mm by 5.6 mm in diameter and 1.8 mm in depth. Slight meningeal artery impressions are visible on the inner table. Vascular channels are present on the ectocranial surface of the frontal. Tooth wear is moderate. Slight osteophyte formation is visible on the superior and inferior endplates of the cervical vertebrae. The femoral heads exhibit compact cancellous bone. The distal joint of the left first metatarsal has slight to moderate arthritic lipping.

Sex

Features consistent with a male sex include a pronounced external occipital protuberance and large mastoid processes. The femora are also robust with well-defined lineae aspera.

Stable Isotope Testing Results

Fragments of the cranium, left humerus, a right metatarsal, as well as the right maxillary third molar, were selected for isotope and heavy metals analysis (Tables S1 and S2). Smithsonian Museum Conservation Institute (MCI) Mass Spectrometry Laboratory followed these procedures for δ^13^C and δ^15^N sample processing:

Samples were run on a Thermo Delta V Advantage mass spectrometer in continuous flow mode coupled to a Costech 4010 Elemental Analyzer (EA) via a Thermo Conflo IV. All calculations of raw isotope values were performed with Isodat 3.0 software. Approximately 0.5mg of tissue were weighed, packed into tin capsules, and introduced to the EA via a Costech Zero Blank Autosampler. Once the N_2_ and CO_2_ molecules were separated, sample gases and reference gas were introduced into the mass spectrometer and regulated by the Conflo IV. The CO_2_ peaks were quantitatively diluted by about 75% for animal tissues. All runs included a set of standards for every 10-12 samples. Standards include Costech Acetanilide and a urea (Urea-UIN3) standard, both of which are calibrated to USGS40 (L-glutamic acid) and USGS41 (L-glutamic acid). All standards were run with the same parameters and procedures as samples. Raw isotope values were corrected using a 2-point linear correction on the calibrated Costech Acetanilide and urea standards. The weight %N and weight %C values were calculated using a peak area calibration based on the homogeneous Costech Acetanilide standard. Reproducibility of standards was ≤0.2‰ (1σ) for both δ^13^C and δ^15^N. The error associated with all sample data points was ±0.2‰.

*taken from*: δ^13^C and δ^15^N Basic Mass Spec Procedures and Parameters (Instrument #1, Costech EA-IRMS), Smithsonian MCI Stable Isotope Mass Spectrometry Laboratory, Christine France 06/15/2015

**Table S1. Stable carbon (collagen) isotope values*.**

| **Burial**  **ID** | **Laboratory** | **Year of Analysis** | **Sample** | **Collagen**  **Yield (%)** | **δ^13^C**  **Collagen (‰)** | **Weight % C** | **C:N** |
| --- | --- | --- | --- | --- | --- | --- | --- |
| **JR2992C** | Paleo-Isochem | 2014 | humerus | 0.6 | -18.1 | 43.8 | 3.3 |
|  |  | 2015 | cranial fragments | 0.7 | -19.0 | 6.1 | 3.5 |
|  | Smithsonian Museum Conservation Institute | 2014 | maxillary third molar | 3.2 | -19.6 | 36.7 | 3.5 |
| **JR170C** | Paleo-Isochem | 2014 | tibia and fibula fragments | 1.4 | -19.7 | 31.3 | 3.6 |
|  | Smithsonian Museum Conservation Institute | 2014 | mandibular first molar | 12.9 | -19.6 | 37.7 | 3.3 |

*****For additional data on sample preparation see France (2015).

**Table S2. ICP-MS testing (Smithsonian’s Museum Conservation Institute).**

| **Sample Type** | **Sample Description** | **Cu** | **Zn** | **As** | **Rb** | **Sr** | **Cd** | **Ba** | **Pb** |
| --- | --- | --- | --- | --- | --- | --- | --- | --- | --- |
| JR2992C | right metatarsal fragments | 4.86 | 292 | 0.71 | 2.37 | 329 | 0.33 | 310 | 147.3 |
| JR170C | left tibia fragments | 3.53 | 148 | 0.42 | 1.99 | 289 | 0.25 | 155 | 126.8 |

*For additional data on sample preparation see Little et al. (2014).

# JR170C

The southern burial in the 1608 Church Chancel was disturbed by an east-west boundary ditch that was filled in by the 1660s (Givens et al. 2016). The ditch overlaid the upper body and legs and damaged the pelvis. Centuries later, in 1938, a hand-dug utility trench severely damaged the skeleton’s right side, removing the right femur and shaving off the anterior left half of the skull. The anterior surface of the left patella was also shaved off.

The individual was buried supine in an east-west aligned anthropomorphic-shaped coffin with the head to the west. The head primarily rested on its right side with the chin down. The iron coffin nails clearly define the anthropomorphic shape of the coffin’s head end. The nails are large and match those found with JR2992C.

The lateral profile of the cranium was defined during the excavation with only the posterior half of the left mandible well-preserved. Several loose teeth are present. A small portion of the distal right radius and ulna are present alongside the torso, indicating placement of the right arm along the side of the body. The left humerus is positioned along the skeleton’s side, as is the left ulna. The left radius was displaced when digging the utility trench.

Clearly visible in the vicinity of the distal left humerus and ribs are black stains. The discoloration reflects the presence of silver thread, the fragments of which are visible on the arm. A metal detector survey indicates that they are present over the chest, as are tiny, silver spangles. The chest region was removed *en block* for laboratory excavation. Following block removal and prior to laboratory excavation, the soil from the chest region was scanned using Xradia Versa 520 X-ray computed tomography instrumentation by Cornell University’s Biotechnology Resource Center. The non-destructive 3D imaging technique recorded in detail the minute silver threads and spangles within the soil matrix (<https://historicjamestowne.org/archaeology/chancel-burials/founders/william-west/> ). These were part of a bundled sash or scarf positioned over the chest of this individual.

The right femur is missing, and the left femur has postmortem breakage. Left hand proximal phalanges are resting on the anterior surface of the proximal left femur. The left patella is resting on the femur’s distal metaphysis. The anterior surface of the left patella was shaved off by the excavation of the utility trench. The tibiae and fibulae are present with the knees and ankles closely spaced.

Crown to heel in situ measurement length is 174 cm; shoulder breadth is 38.2 cm; leg length is 91 cm (Lt), and the femur length is 48 cm (Lt). The breadth across the knees as measured from the outsides of the tibiae metaphyses is 16.8 cm. The outside breadth at the ankles is 16.6 cm. This is the tallest individual of the four Chancel men.

The skeleton removed from the field is highly fragmented and poorly preserved. However, the long bone cortices are not as degraded as observed for the other three burials. This may be a product of the individual’s younger age and thicker cortical bone. Vertebrae are present, but partially represented by centra and neural arches. Thoracic vertebrae eleven and twelve, and lumbar vertebrae one through five are represented by articular facets only. Only one rib fragment is present. No joint surfaces are present for any of the long bones, except for the metatarsals and partial left femoral head. Green cuprous staining, confirmed using XRF analysis, is present on the internal surface of the right ilium.

All right maxillary teeth are present. The right first molar has a small occlusal cavity. No apical abscesses are present. The maxillary teeth show slight wear. The left side maxillary dentition is represented by a loose third molar.

The mandible is nearly complete with postmortem breakage. The right mandibular dentition is represented by the canine, first premolar, and first, second and third molars. The right second premolar was lost antemortem and the alveolar socket had remodeled with slight posterior drift of the first premolar and slight medial drift of the first molar. Trace occlusal wear is noted, with the exception of the first molar, which has beginning dentin exposure. The left mandibular dentition is represented by the second premolar, and first, second and third molars. The anterior sockets are present but empty due to postmortem tooth loss.

Age

This skeleton is the youngest of the four chancel burials. An age of 22 to 25 years is based on tooth wear and tooth root formation. The left maxillary third molar has Apex-1/2 formation of the mesial buccal root. This opening could also be a remnant opening retained into the 20s. However, tooth wear is slight with no wear noted on the third molars. The C1 dens facet has a sharp margin. The C2 dens facet has no lipping. The cervical and first thoracic epiphyseal rings are fused. Remnant lines of union are still visible for some of the cervical epiphyseal rings. The cervical vertebrae have no arthritic changes. Epiphyses are fused where observations of epiphyseal formation are possible (e.g., left distal fibula and distal tibia). The partially preserved right auricular surface exhibits subtle retention of surface billowing at the superior margin. A left ilium fragment exhibits fusion of the iliac crest. The cortical bone is dense, and cortical surfaces are smooth. Cancellous bone spacing is compact (e.g., the left proximal tibia and left proximal and distal femur exhibit compact cancellous bone). The left femur and tibia have smooth external cortices, with only initial formation of longitudinal striae. The linea aspera, although defined, lacks rugosity that is more characteristic of older adults. No arthritic changes are observed on the calcanei, tali, or left first metatarsal.

Sex

The mandible is large. The gonial angle is acute and the body is thick with a square mental eminence. The long bones are robust and large. The right auricular surface is not raised. All features are consistent with a male identification.

Pathology

The L4 exhibits an incomplete fracture along the inferior marginal rim of the left inferior facet. A piece of the rim measuring 7.7 mm (transverse) by 4.5 mm (superior-inferior) is partially detached, leaving a line of separation that has subsequently remodeled.

Stable Isotope Testing Results

Fragments of left tibia and a left mandibular first molar were tested for isotopes and heavy metals (Tables S1 and S2).

**OSM2: Ancient DNA Methods**

*Data Generation*

Following UV irradiation and surface removal with a sanding disk to remove any contamination on the surface, ~75mg of bone powder was sampled from a petrous temporal of both individuals using a Dremel drill. Individual JR170C required additional sampling from a tooth extracted from the mandible due to extremely poor DNA preservation in the petrous. No analyzable DNA was generated from the original petrous sample for this individual; therefore results are only reported from the second round of sampling (Table S3).

DNA extraction was performed according to a previously described protocol (Rohland *et al.* 2018) using binding buffer D. Library preparation was performed using either a single or double stranded preparation method with either no (Meyer & Kircher 2010; Kircher 2012) or partial-UDG treatment (Rohland *et al.* 2015; Gansauge *et al.* 2020). Targeted enrichment capture was used to enrich for the entire mitochondrial genome and ~1.2 million sites in the nuclear genomes that are informative for population genetic analyses (Fu *et al.* 2013; Fu *et al.* 2015; Haak *et al.* 2015; Mathieson *et al.* 2015). Sequencing was performed on an Illumina NextSeq500 sequencer with 2x75 cycles in addition to 2x7 indexing cycles.

Sequencing reads were then merged by individual, matching the 7 base pair indices at the 5’ and 3’ ends of each molecule and allowing for no more than one mismatch per index/barcode. Sequencing adapters were removed using the tool SeqPrep (<https://github.com/jstjohn/SeqPrep>). Paired end reads were then merged, requiring a minimum of 15 base pair overlaps, with up to 1 mismatch. Next, sequence reads were aligned to the RSRS mitochondrial genome (Behar *et al.* 2012) or to the hg19 human reference genome, using samse in BWA (v0.6.1) (Li and Durbin 2009). Duplicate sequences were then removed by filtering out reads with identical start and end positions, orientation, and barcode pairs, retaining only the highest quality sequence for each duplicate. Since there was not sufficient coverage to call diploid genotypes, pseudo-haploid genotypes were called at each SNP position for the ~1.2 million SNP positions targeted during enrichment capture. At each position, a randomly chosen sequence covering each targeted site was selected to represent the pseudo-haploid genotype after stripping either two or ten bases at each end of the molecule, for libraries prepared using a partial UDG or no UDG treatment, respectively, and restricting to sites with reads having a minimum mapping quality (MAPQ>10) and base quality (>20).

The ratio of Y chromosome to X chromosome sequences was used to determine molecular sex (Skoglund *et al.* 2013). Next, close genetic relatives (3^rd^ degree or closer) were identified using an approach that measures the relative rate of allele sharing between individuals, as described in Supplementary Information 6 in Olalde *et al.* (2019).

Haplogrep2 (Weissenstiner *et al.* 2016) was used to determine mitochondrial haplogroups. The mitochondrial consensus sequence was generated from reads aligning to the rsrs mitochondrial genome by trimming either 10 or 2 base pairs from the terminal ends of each read for libraries that underwent no damage correction (i.e., “minus”) or partial damage correction (i.e., “half” or “USER”), respectively. Haplogroup assignments and quality scores are reported in Table S4. Y-chromosome haplogroups were determined following a procedure described in Supplementary Text 5 of Lazaridis *et al.* (2022) that considers all sequences that aligned to the Y chromosome (Table S5). The Yfull 8.09 tree ([https://www.yfull.com/)](https://www.yfull.com/)d) was used during calling and Y chromosome haplogroup was denoted using the ISOGG notation (version 15.73, http://isogg.org).

Two standard methods were used to assess the authenticity of the sampled ancient DNA. First, contamMix (Fu *et al.* 2013) was used to determine the rate of matching to the consensus sequence in the mitochondrial genome. 97% was considered the minimum acceptable threshold of authenticity. Next, the rate of C-to-T substitutions at the terminal ends of DNA molecules was measured. Thresholds of 10% and 3% were considered to be the minimum threshold for authenticity in libraries prepared using either no or partial-UDG treatment, respectively (Rohland *et al.* 2015). There was not sufficient sequencing coverage to assess the rate of contamination in the X-chromosome for genetically male individuals, as is typically done using the tool ANGSD (Korneliussen *et al.* 2014), as this requires a minimum of 200 SNPs sequenced on the X-chromosome.

*Analysis Dataset*

The tool mergeit (Patterson *et al.* 2006) was used to combine the newly reported historical data with published data from 68 present-day populations (Patterson *et al.* 2012; Lazaridis *et al.* 2014; Lazaridis *et al.* 2016; Jeong *et al.* 2019; Biagini *et al.* 2019). The resulting working dataset contained a total of 597,573 SNPs. All subsequent population genetic analyses were performed after restricting to transversion SNP positions to avoid biases caused by the mixed library preparation types.

*Principal Component Analysis*

Principal component analysis (PCA) was performed using the program *smartpca* (Patterson *et al.* 2006). The historical individuals were projected onto a PCA background produced using data of 1320 present-day individuals from 68 European and Near Eastern populations (Lazaridis *et al.* 2016) to explore their West Eurasian ancestry. Default parameters were used with the settings lsqproject:YES, shrinkmode:YES, and number outlier:0. Additionally, 95% confidence ellipses were calculated for the four historic individuals using ellconf: 0.95.

**Table S3. Expanded information on the ancient DNA analysis**

|  | | | | **Mitochondrial DNA** | | | **1.15 million autosomal targets** | | **Library information** | | | | |
| --- | --- | --- | --- | --- | --- | --- | --- | --- | --- | --- | --- | --- | --- |
| Lab ID | Skeletal element | Genetic sex | Y-chrom. haplogroup | Average coverage | haplogroup | Match to consensus (%) | Average coverage | Unique SNPs | No. libs. | Library ID | Library type* | Fraction human (shotgun data) | Damage first nucleotide** |
| I2096 | petrous | M | I1 | 17 | H10e | 98.0 ± 1.0% | 0.069 | 76449 | 6 | S4819.E1.L1 | ds.minus | 0.0059 | 0.162 |
|  |  |  |  |  |  |  |  |  |  | S2096.E1.L1 | ds.half | 0.0008 | n/a |
|  |  |  |  |  |  |  |  |  |  | S2096.E1.L2 | ss.USER | 0.00159 | 0.267 |
|  |  |  |  |  |  |  |  |  |  | S2096.E1.L3 | ss.USER | 0.00141 | 0.249 |
|  |  |  |  |  |  |  |  |  |  | S2096.E1.L4 | ss.USER | 0.00166 | 0.271 |
|  |  |  |  |  |  |  |  |  |  | S2096.E1.L5 | ss.USER | 0.00139 | 0.27 |
| I4652 | tooth | M | F | 161 | H10e | 99.8 ± 0.1% | 0.011 | 12657 | 3 | S4652.E1.L1 | ds.half | 0.0021 | 0.023 |
|  |  |  |  |  |  |  |  |  |  | S4652.E1.L4 | ss.USER | 0.00023 | 0.114 |
|  |  |  |  |  |  |  |  |  |  | S4652.E1.L5 | ss.USER | 0.00028 | 0.116 |

* Library type: minus = no damage correction, half or USER = damage retained at last position, ds = double- stranded, ss = single-stranded)

** Damage rate in first nucleotide computed on sequences mapping to the human genome.

**Table S4. Mitochondrial haplogroup calls and mutations.**

| **Lab ID** | **Assigned Mitochondrial Haplogroup** | **Quality** | **Mutations Missing** | **Mutations Present** | **Mutations Remaining** |
| --- | --- | --- | --- | --- | --- |
| I2096 | H10e | 0.9366 | 1438G | 263G 750G 4769G 8860G 14470A 15326G 16093C 16221T | 3106d 16519C |
| I4652 | H10e | 1 |  | 263G 750G 1438G 4769G 8860G 14470A 15326G 16093C 16221T | 310d 311d 312d 313d 3106d 16519C |

**Table S5. Y haplogroup calls and mutations**

| **Lab ID** | **I2096** | **I4652** |
| --- | --- | --- |
| **Assigned Y Haplogroup** | I1 | F |
| **Mutations Present** | I1:CTS11042(22914378T>C:C),CTS11126(22967741C>G:G),CTS1739(14042842A>G:G),Y1871(8349674C>A:A),Y1872(8349675G>C:C),Z2865(22948591A>G:G) | F:CTS3996(15323154A>G:G),F3512(23729951C>T:T) |
| **Mutations Absent** | I-Y7282:Y7283(8873762C>T:C),I-Y18311:Y18316(19281094C>T:C),I-A8585:A8587(15499895A>G:A),I-Y15947:Y15947(6891284C>T:C),I-BY169301:BY170530(17368168G>T:G),I-Y3549:CTS11651(23203680C>G:C),I-Y31032:Y32101(8493063C>A:C),I-Y132292:Y87144(8511720G>A:G),I-PH497:BY86997(13649194G>A:G),I-Y139735:Y139736(16836773C>T:C),I-Y133595:BY34378(16771616C>T:C),I-PH2510:A14166(17314167C>T:C),A14173(23053434G>A:G),I-A5734:Y15882(15778437A>G:A),I-Y23119:Y23126(18163801C>G:C),I-A11537:A11537(17646827G>A:G),I-Y14344:Y14540(24469839G>A:G),I-FT110841:Y43625(21938186A>G:A),I-Y61868:Y36777(7638932C>T:C),I-FGC31738:FGC31738(13676739T>C:T),I-Y17387:Y17703(6636281A>G:A),I-Y7059:Y7060(6952551G>T:G),I-Y11539:BY190333(9384780A>G:A),I-Y29668:A11259(17003176C>T:C),I-Y40258:A6686(16954364C>T:C),I-Y61915:FT244584(22806967C>A:C),I-A13745:A13762(22516842C>T:C),I-FT50952:FT50086(14026457C>A:C),I-A21841:A21845(8524932G>A:G),I-Y73200:BY87414(13660615G>A:G),I-Y36041:A17347(9446004C>T:C),I-Y26079:BY173428(14320075G>A:G),Y26079(22058342G>A:G),I-Y56390:A9272(6932183T>A:T),I-Y22033:Y22033(17888972G>A:G),I-Y31033:Y31035(16760815T>C:T),I-Y4870:FGC36094(14028861G>T:G),I-Y21954:Y22408(17511419C>T:C),I-Y13046:Y13468(8598383C>T:C),I-Y37104:Y37104(8588402C>A:C),I-FT206938:FT206939(8674744A>G:A),FT207500(17309454C>A:C),I-Y15584:FGC23811(7286109A>C:A),I-A9239:A9244(9418013C>T:C),I-A8596:A8603(18727668G>A:G),I-PH4462:PH4523(19474975G>C:G),I-A12708:A12715(22677556A>G:A),I-A1550:A1550(7891602C>T:C),I-Y13025:A1515(21225357T>G:T),FGC23286(21251228C>T:C),I-Y6356:Y6361(15346238C>T:C),I-Y7279:A5924(7873679C>T:C),Y7396(15715950A>G:A),I-Y32666:A14325(8426470G>T:G),I-YP1081:FGC53720(17381683G>A:G),I-Y11205:FT34426(14677834G>T:G),I-A11062:A11063(7074059C>G:C),I-Y21293:A8283(2683462G>A:G),I-S2308:Z17931(8530294C>T:C),I-Y154876:A22348(16729579C>G:C) | G-Y14914:Y17738(4136702A>T:A),G-M286:Z43077(8334942T>C:T),G-S10458:Y19947(23975898C>T:C),G-Y36001:FGC65129(24005520C>T:C),Y36608(6983613G>A:G),G-S20738:Z38840(8848675G>A:G),G-Y164210:BY219823(22446375T>C:T),G-PH488:PH4240(19113971G>A:G),G-M283:FT307855(21562041G>A:G),J:PF4598(22118776A>G:A),I:CTS7593(17548890G>A:G),Y1934(8504226G>A:G),T-M4538:FGC63093(13672284G>A:G),T-M70:CTS1315(7333273C>A:C),L-PAGE116:FGC36863(19485221A>G:A),L-Y31183:FGC63387(8150585G>A:G),Y31208(23282417C>A:C),L-Z5924:Z20470(23314769G>A:G),L-Y60258:Y70107(16549344G>A:G),N:CTS1653(14006122C>T:C),O:CTS7942(17756426A>T:A),K-Y28299:Y28360(13921304G>T:G),M-Z31022:Z31149(22864231C>A:C),S-M230:SK1894(16256100C>T:C),Y27735(8399812C>G:C),Y27813(22188679C>T:C),R2:F3302(22179053G>A:G),R-Y161894:BY195408(18583375T>C:T),R-BY100583:YP443(6994855C>T:C),R-BY191239:BY190826(15802203G>T:G),R-YP1337:FGC32031(17590963T>C:T),R-FGC64132:Y130231(22653282T>C:T),R-YP703:Y23853(13832245G>A:G),R-YP3932:FT81714(6614554G>C:G),R-Y34286:Y34301(8353525G>A:G),R-YP5544:YP5549(7779503G>A:G),R-Y878:Y882(6381834A>T:A),R-Y147630:Y148931(16442195G>T:G),R-CTS6:CTS6(2657349T>C:T),R-FGC14376:FT261742(19113989T>C:T),R-Y19434:Y19434(14284667C>T:C),R-Y157951:BY180505(15033004A>G:A),R-Y107553:Y90917(14280442T>C:T),R-Y29937:Y29937(17884866G>T:G),R-FGC57007:FGC57012(17909243C>G:C),R-Y145978:Y145978(17729362G>A:G),R-Y20966:Y21433(15548870A>G:A),R-Y5113:Y5122(17052212T>G:T),R-Z278:Z273(22483650C>T:C),R-Y31385:Y31385(15323142C>T:C),R-BY30439:BY30438(23023463T>G:T),R-A9850:A9857(19287786C>A:C),R-Y20986:Y20988(23986241C>T:C),R-Y86996:Y110085(22906677C>T:C),R-A11676:A11676(8848634C>T:C),R-Y101215:Y106163(21332260G>A:G),R-Y19742:S1555(2888588T>C:T),R-L196:A1736(23060400A>T:A),R-CTS9462:CTS9462(18907973C>T:C),R-BY150850:Y143805(8465915G>A:G),R-Y32819:Y32818(7583477A>G:A),R-Y66080:Y66528(14345712G>A:G),R-ZP85:Y16119(9386909T>C:T),R-FGC21155:FGC21151(7128625T>C:T),R-Y19736:Y19735(4136677C>T:C),R-FGC54632:BY85974(13477413G>T:G),R-BY13963:Y32860(21642899G>A:G),R-Y19118:Y19121(19096771C>A:C),R-FGC23826:Y18881(6918883C>T:C),R-A10127:A10152(22817958A>T:A),R-PH155:BY14355(8400716A>G:A),Q-L275:Y1102(19524650C>A:C),Q-Y6794:SK1937(19147027A>G:A),Q-L940:F1513(9172578G>A:G),Q-CTS2731:YP4720(23568943G>T:G),Q-YP4722:MPB161(18128727G>A:G),Q-Y32170:BY15683(8466593T>A:T),BY15684(8466598A>C:A),BZ4454(8466578C>A:C),BZ4455(8466579T>A:T),Q-BZ1700:FGC12250(17590973A>G:A),Q-YP910:YP914(17383880G>C:G),Q-Z782:CTS9614(18990978G>A:G),Q-YP4004:Y15808(6381801A>G:A),H-BY40831:BY44426(21507251C>T:C),H-Z5862:Z12675(17754902C>T:C),H-M8081:Z13810(21332253G>T:G),H-M69:M370(2888598C>G:C) |

**References**

1000 Genomes Project Consortium. 2015. A global reference for human genetic variation. *Nature* 526:68–74.

Biagini, S.A., N. Solé-Morata, E. Matisoo-Smith, P. Zalloua, D. Comas, & F. Calafell. 2019. People from Ibiza: an unexpected isolate in the Western Mediterranean. *European Journal of Human Genetics* 27(6):941–951.

Behar, D.M., M. van Oven, S. Rosset, M. Metspalu, E.-L. Loogväli, N.M. Silva, T. Kivisild, A. Torroni, & R. Villems. 2012. A “Copernican” reassessment of the human mitochondrial DNA tree from its root. *The American Journal of Human Genetics* 90(4):675–684. doi:10.1016/j.ajhg.2012.03.002.

Buikstra, J.E., & D.H. Ubelaker (eds.). 1994. *Standards for Data Collection from Human Skeletal Remains.* Arkansas Archaeological Survey, Fayetteville.

Delano, J.W. 2022. Eastward orientations of 17^th^ –19^th^ century churches in the eastern United States: A potential source of chronological information. Draft copy dated June 14, 2022 on file at the National Museum of Natural History, Department of Anthropology. Submitted to *Post-Medieval Archaeology.*

France, C. 2015. Stable isotope analysis of human remains from Jamestown Colony. Data report, Smithsonian Museum Conservation Institute, Suitland, Maryland.

France, C.A.M., D.W. Owsley, K.S. Bruwelheide, E.S. Renschler, K.G. Barca, & C.R. DeCorse. 2019. Stable isotopes from the African site of Elmina, Ghana and their usefulness in tracking the provenance of enslaved individuals in 18^th^- and 19^th^-century North American populations. *American Journal of Physical Anthropology* 171(2):298–318. doi:10.1002/ajpa.23946.

Fu, Q., M. Meyer, X. Gao, U. Stenzel, H.A. Burbano, J. Kelso, & S. Pääbo. 2013. DNA analysis of an early modern human from Tianyuan Cave, China. *Proceedings of the National Academy of Sciences* 110(6):2223–2227. doi:10.1073/pnas.1221359110.

Fu, Q. *et al*. 2015. An early modern human from Romania with a recent Neanderthal ancestor. *Nature* 524:216–219. doi:10.1038/nature14558.

Gansauge, M.-T., A. Aximu-Petri, S. Nagel, & M. Meyer. 2020. Manual and automated preparation of single-stranded DNA libraries for the sequencing of DNA from ancient biological remains and other sources of highly degraded DNA. *Nature Protocols* 15:2279–2300. doi:10.1038/s41596-020-0338-0.

Givens, D., L. Fischer, J. Horn, W. Kelso, K. Bruwelheide, & D. Owsley. 2016. *Holy Ground: Archaeology, religion, and the first founders of Jamestown.* The Jamestown Rediscovery Foundation and Preservation Virginia.

Haak, W. *et al.* 2015. Massive migration from the Steppe was a source for Indo-European languages in Europe. *Nature* 522:207–211. doi:10.1038/nature14317.

Jeong, C. *et al*. 2019. The genetic history of admixture across inner Eurasia. *Nature Ecology & Evolution* 3:966–976. doi:10.1038/s41559-019-0878-2.

Kircher, M. 2012. Analysis of high-throughput ancient DNA sequencing data. *Methods in Molecular Biology* 840:197–228. doi:10.1007/978-1-61779-516-9_23.

Korneliussen, T.S., A. Albrechtsen, & R. Nielsen. 2014. ANGSD: Analysis of next generation sequencing data. *BMC Bioinformatics* 15:356. doi:10.1186/s12859-014-0356-4.

Lazaridis, I. *et al*. 2014. Ancient human genomes suggest three ancestral populations for present-day Europeans. *Nature* 513(7518):409–413. doi.org/10.1038/nature13673.

Lazaridis, I. *et al*. 2016. Genomic insights into the origin of farming in the ancient Near East. *Nature* 536(7617):419–424. doi.org/10.1038/nature19310.

Lazaridis, I. et al. "The genetic history of the Southern Arc: A bridge between West Asia and Europe." *Science* 377.6609 (2022): eabm4247. https://www.science.org/doi/abs/10.1126/science.abm4247

Li, H., & R. Durbin. 2009. Fast and accurate short read alignment with Burrows–Wheeler Transform. *Bioinformatics* 25(14):1754–1760. doi:10.1093/bioinformatics/btp324.

Little, N.C., V. Florey, I. Molina, D.W. Owsley, & R.J. Speakman. 2014. Measuring heavy metal content in bone using portable x-ray fluorescence. *Open Journal of Archaeometry* 2(5257): 19–21. doi:10.4081/arc.2014.5257.

Mathieson, I. *et al*. 2015. Genome-wide patterns of selection in 230 ancient Eurasians. *Nature* 528:499–503. doi:10.1038/nature16152.

Meyer, M., & M. Kircher. 2010. Illumina sequencing library preparation for highly multiplexed target capture and sequencing. *Cold Spring Harbor Protocols* doi:10.1101/pdb.prot5448.

Olalde, I. *et al*. 2019. The genomic history of the Iberian Peninsula over the past 8000 years. *Science* 363:1230–123. doi:10.1126/science.aav4040.

Patterson, N., P. Moorjani, Y. Luo, S. Mallick, N. Rohland, Y. Zhan, T. Genschoreck, T. Webster, & D. Reich. 2012. Ancient admixture in human history. *Genetics* 192(3):1065–1093. doi:10.1534/genetics.112.145037.

Patterson, N., A.L. Price, & D. Reich. 2006. Population structure and eigenanalysis. *PLoS Genetics* 2:e190. doi:10.1371/journal.pgen.0020190.

Rohland, N., I. Glocke, A. Aximu-Petri, & M. Meyer. 2018. Extraction of highly degraded DNA from ancient bones, teeth and sediments for high-throughput sequencing. *Nature Protocols* 13:2447–2461. doi:10.1038/s41596-018-0050-5.

Rohland, N., E. Harney, S. Mallick, S. Nordenfelt, & D. Reich. 2015. Partial uracil–DNA–glycosylase treatment for screening of ancient DNA. *Philosophical Transactions of the Royal Society B* 370:20130624. doi:10.1098/rstb.2013.0624.

Skoglund, P., J. Storå, A. Götherström, & M. Jakobsson. 2013. Accurate sex identification of ancient human remains using DNA shotgun sequencing. *Journal of Archaeological Science* 40(12):4477–4482. doi:10.1016/j.jas.2013.07.004.

Skoglund, P., B.H. Northoff, M.V. Shunkov, A.P. Derevianko, S. Pääbo, J. Krause, & M. Jakobsson. 2014. Separating endogenous ancient DNA from modern day contamination in a Siberian Neandertal. *Proceedings of the National Academy of Sciences* 111(6):2229–2234.

doi:10.1073/pnas.1318934111.

Ubelaker, D.H., & D.W. Owsley

2003 Isotopic evidence for diet in the seventeenth-century colonial Chesapeake. *American Antiquity* 68(1):129–139.

Weissensteiner, H., D. Pacher, A. Kloss-Brandstätter, L. Forer, G. Specht, H.-J. Bandelt, F. Kronenberg, A. Salas, & S. Schönherr. 2016. HaploGrep 2: Mitochondrial haplogroup classification in the era of high-throughput sequencing. *Nucleic Acids Research* 44(W1):W58–W63. doi:10.1093/nar/gkw233.
